# Modern Medico-Psychology and Psychiatry

**Published:** 1895-03-09

**Authors:** T. S. Clouston

**Affiliations:** Lecturer on Mental Diseases, Edinburgh University, and Physician Superintendent Royal Asylum, Morningside


### MODERN MEDICO-PSYCHOLOGY AND PSYCHIATRY.

By T. S. CLOUSTON, M.D., Lecturer on Mental Diseases, Edinburgh University, and Physician Superintendent Royal Asylum, Morningside.

### STATES OF FIXED DELUSION.

The typical form of insanity, in the lawyer's mind, is that accompanied by insane delusion. Delusion is, in fact, his chief test and proof of legal unsoundness of mind. In the doctor's estimation it holds no such place of prominence so far as diagnosis and treatment are concerned. To him it is only one of a series of mental symptoms. Some one bodily symptom in a case may lead him to far more important conclusions as to recovery or treatment than a delusion. A tremulous shiver about the lips, accompanying a minute slurring of speech, may tell him far more of the brain-condition of a patient than any delusion. Psychologically, delusions are exceedingly interesting because they are interferences with the highest mental faculty—that of reasoning. When a man cannot, through a disease of his brain, come to right conclusions on simple matters of judgment, however clear the premisses may be; when he appears to receive absolutely wrong impressions from his senses, his organs of sense being in a sound condition, all the calculations of the psychologist are upset. He must fall back on the theory of a disordered instrument of mind, because there is no conceivable theory of mind pure and simple that could possibly explain the facts.

Delusion is a term in everyone's mouth, applied to all sorts of belief of others which the speaker believes to be mistaken; all sorts of errors in reasoning, and of ignorances, are popularly and in literature called delusions. All sorts of superstitions are rea delusions, but not insane delusions. Anyone who wants to realise what an insane delusion is like to the person who labours under it, has merely to think of his last dream. Vivid dreams and nightmares are, in fact, the strict physiological counterpart of pathological insane delusions, and they sometimes arise in the brain from the same cause. A psychiatrist always looks first for a bodily cause for a delusion. I had a woman who affirmed to me that the devil was inside her, and pointed to a particular spot over her stomach where she felt him. In time we discovered a growing cancer there. Her sensations were real; it was a false interpretation of them that constituted the insane delusion. Have we not all in nightmare, after having dined not wisely but too well, had the belief that heavy men sat on our stomachs, whose presence was vividly real to what was then active of our minds? The cerebral mental cortex of my patient was disordered in working by disease. Our cerebral cortex during the nightmare was partially suspended in its action by sleep, and it misinterpreted the real discomfort the undigested food was causing into a delusion. Every organ and every function is represented in the brain, and has a "centre" there for its control and regulation. There are three ways of seeing a light. One is by the presentation of the real light to the eye, and the next is by striking the eye, and the third is by stimulating the visual centre in the brain cortex. The lights presented to consciousness by the two latter methods are of the same nature as some insane delusions. I have a woman who complains to me every morning that men come and smash and break her legs and fingers every night when she is asleep. In reality, she has rheumatic pains in her legs and fingers which excite the centres of common sensation of those limbs in the brain during sleep, and she is unable by correct reasoning to attribute these real sensations to their true cause. That disturbance in the reasoning parts of the brain, the comparing and judging portions, constitutes her unsoundness of mind. The discovery of a bodily cause for a delusion has the enormous advantage that it may then possibly be treated and removed, and the brain irritation may gradually subside. Very few insane delusions indeed are benefitted in the least by reasoning about them and demonstrating their falsity; commonly they are strengthened thereby. But as the brain is recovering from its pathological disturbance, then a demonstration that a belief is unreal may come in usefully as a therapeutic measure. I had a patient lately who had drunk and hard developed two insane delusions in consequence. One being that his food was poisoned, and the other that men annoyed him at night calling him foul names. He "heard their voices" when he went to bed every night. Knowing that the mucous membrane of his stomach was in a state of irritation from the quantity of bad whisky that he had constantly put into it, and that his brain cortex was in a state of irritation from the same whisky after it had been absorbed, no more stimulants were given; very light, unstimulating diet, only in small quantities at a time, was given him, with remedies that soothed the irritable gastric membrane. Some bromides were given to soothe the irritated auditory centres in his brain, while his general health was improved by exercise and right living. At first no demonstration that his food was wholesome and that he could take it with impunity, or that no men could possibly be speaking through the walls of his room were of any avail to dispel his delusions, but, as his stomach got put to rights, he gradually ceased to harbour the poisoning delusion, and then, more slowly, he ceased to hear the voices when the effects of the alcohol disappeared from his brain.

Such cases show the present medical view of insane delusions. We always ask, Is there any outside or inside source of irritation that can be removed, or can the irritable brain-centres be soothed and put to rights? Most cases of insanity have delusions of some sort. Some of these are transitory and ever-changing as in acute mania; some are more permanent, but not quite fixed, as is the case mostly in melancholia; some are quite fixed, as in monomania and paranoia. The quality of the delusions is commonly determined by the emotional state of the patient. In hilarious mania they are joyous, in suicidal melancholia they are painful. We do not like very fixed delusions, unchanging in character from month to month, especially if they have developed slowly and gradually. Bodily diseases that lower the strength, like consumption, are often accompanied by painful delusions of suspicion. Fixed delusions apart from general exaltation or excitement or depression are not favourable as regards recovery.

In a certain sense the undeveloped brain in early childhood, before the connections so recently demonstrated by Flechsig, are fully made between the sense centres and the true mental centres in the cortex, exhibits nothing but delusions. No facts are truly observed, no true conclusions are come to by any exercise of reason, while the joyous fancies, the castles in the air, and the whole mode of regarding the outside world, are mostly unreal and delusional. There is the closest scientific analogy between the joyous delusions of the evolving brain of the child of two, and the exalted ideas of the general paralytic evolved through the dissolution of his brain by disease.

